# Supplementary material for: Catalytically controlled formation of coumarin-based hydrogelator enables colorimetric ferrous ion detection in sol and hydrogel
Source: Commun Chem. 2025 Nov 26;8:372. doi: 10.1038/s42004-025-01760-3 (PMC12658006; doi:10.1038/s42004-025-01760-3)
Supplement: Supplementary file 2 — Supplementary Information [file 42004_2025_1760_MOESM2_ESM.pdf]

Supporting Information for

Catalytically Controlled Formation of Coumarin-based Hydrogelator Enables Colorimetric Ferrous Ion detection in Sol and Hydrogel

Nikita Das[1], Samir Mandal[2], Sib Sankar Mal[3], Suryasarathi Bose[2], Chandan Maity*[1]

[1](Organic)Materials and Engineering Laboratory, Centre for Nanobiotechnology (CNBT), Vellore Institute of Technology (VIT), Tamil Nadu-632014, India.

[2]Department of Materials Engineering, Indian Institute of Science, Bangalore-560012, India

[3]Materials and Catalysis Laboratory, Department of Chemistry, National Institute of Technology Karnataka, Surathkal-575025, India

Email: chandanmaitylab@gmail.com and chandan.maity@vit.ac.in

1. Materials

All chemicals were commercially available and were used as received without further purification. 4-chloro-3-formylcoumarin was purchased from *Sigma Aldrich*, guanidine hydrochloride from *Tokyo Chemical Industry (TCI)*, hydrazine hydrate from *Central Drug House (CDH) Pvt. Ltd.* HEPES buffer was obtained from *Sisco Research Laboratories (SRL) Pvt. Ltd*. All solvents for NMR measurement were purchased from *Sigma Aldrich*. Methanol, DMSO, DMF, THF used are of spectroscopic grade. For sensing purpose, nitrate salts of all cations were used. Deionised water was used for all experiments (if required).

2. Experimental section

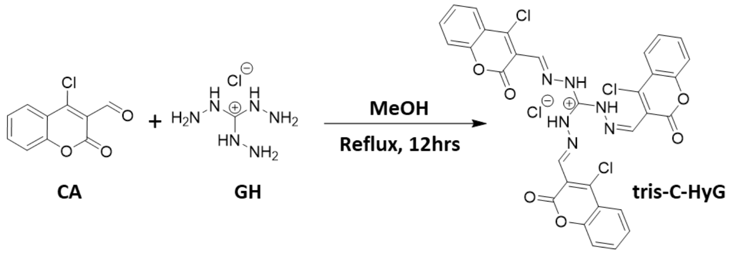

Scheme S1: *Synthesis of hydrogelator* ***C-HyG*** *via the condensation of 4-chloro-3-formylcoumarin (CA) and tris-hydrazide (GH).*

- **Synthesis of coumarin based hydrogelator (C-HyG)**

Guanidine-tris-hydrazine (GH) was synthesized using reported procedure.[S1] C-HyG was prepared as outlined in Scheme S1. 4-chloro-3-formylcoumarin (CA) (0.624 g, 3.0 mmol) and GH (0.14 g, 1.0 mmol) were dissolved in methanol (20 mL) with few drops of acetic acid. The mixture was refluxed for 12 hours. Then, it was allowed to cool to room temperature, resulting in the formation of a gelatinous brown precipitate. The precipitate was filtered, and washed with a solvent mixture (10.0 mL, methanol: water in 9:1 ratio) to remove any unreacted starting materials. The resulting precipitate was dried under reduced pressure at room temperature and characterised via ^{1}H NMR, HRMS and FTIR spectroscopy.

^{1}H NMR (400 MHz, DMSO) δ = 8.47 (s, 3H, C-H associated in hydrazone bond), 8.05 (d, *J* = 7.8 Hz, 3H), 7.76 (d, *J* = 7.9 Hz, 3H), 7.52 (d, *J* = 6.7 Hz, 6H). HRMS (ESI Pos) m/z: 677.03 [$(M)^+$], 699.01 [$(M\text{-}H + Na)^+$].

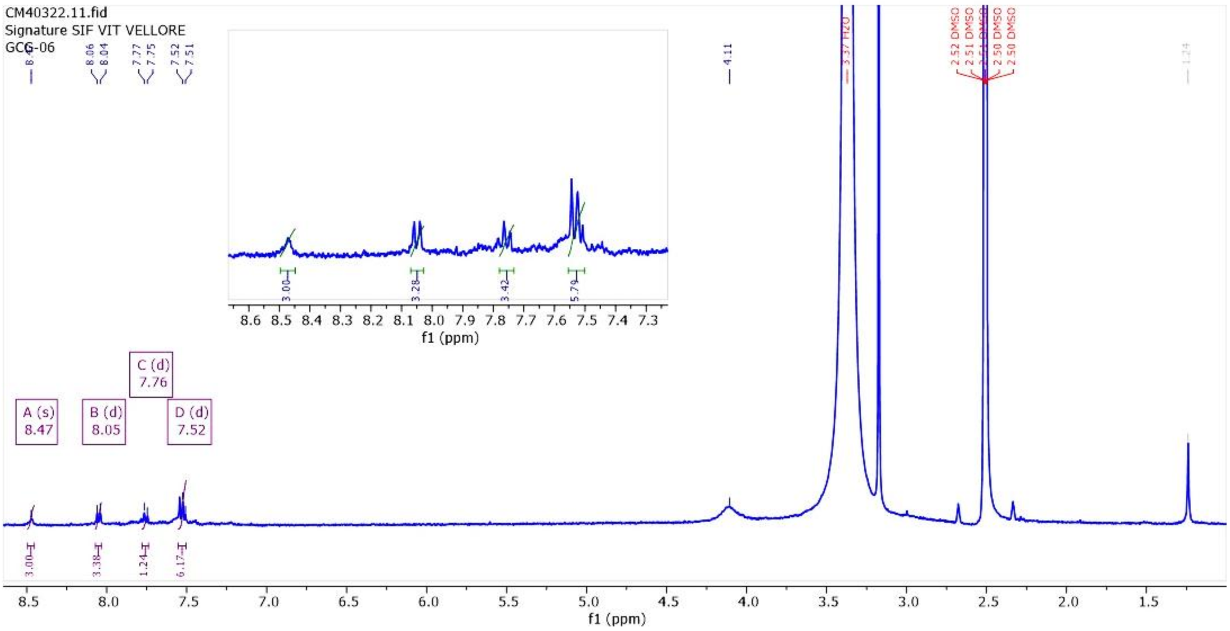

***Figure S1**: ^{1}H NMR spectra of gelator **C-HyG** in DMSO-D_6.*

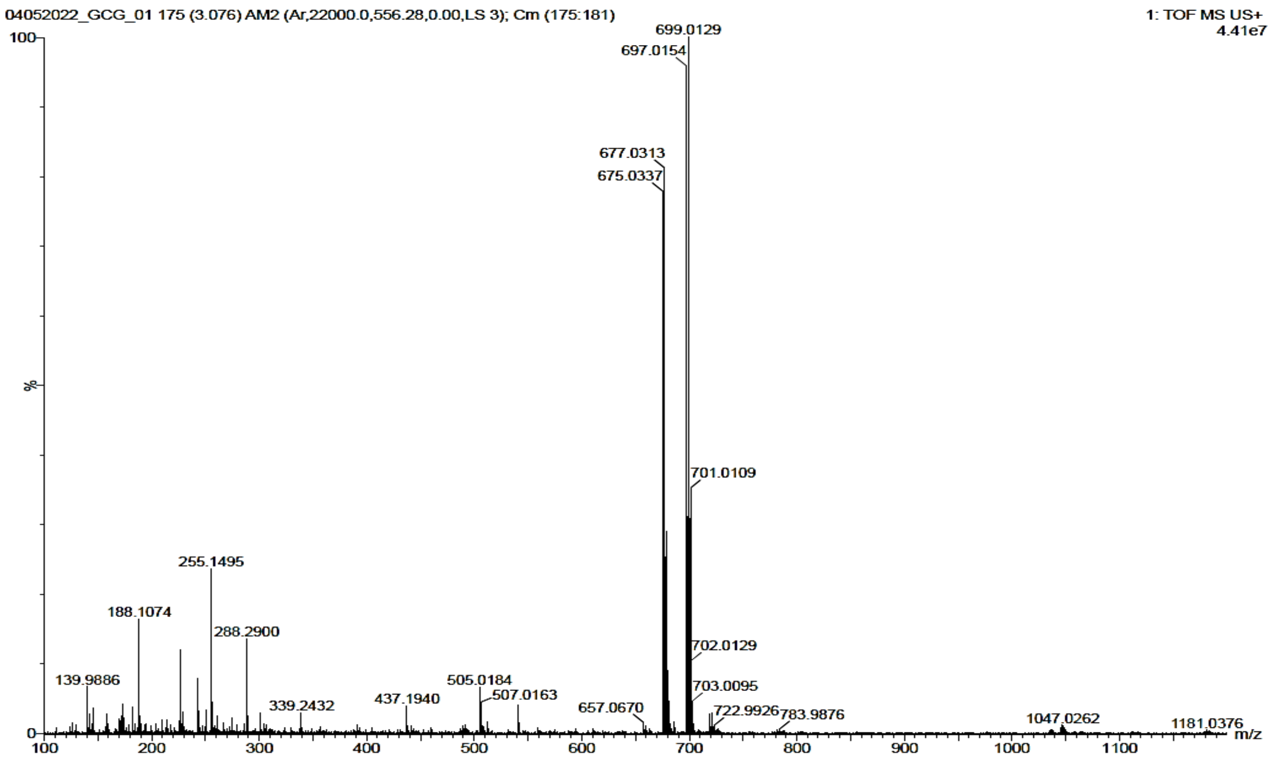

Figure S2: *HRMS spectrum of the hydrogelator* ***C-HyG****.*

- **FTIR analysis**

The FTIR spectrum of C-HyG (green line, Figure S3) exhibited a very broad peak at 3508 cm^{-1} and a sharp peak at 1720 cm^{-1}, corresponding to imine (C=N) stretching. In contrast, the spectrum of CA (blue line, Figure S3) showed characteristic peaks at 1610 cm^{-1} (C=O stretching) and 842 cm^{-1} (C–Cl stretching). Additionally, GH (red line, Figure S3) displayed sharp primary amine peaks in the region of 3400–3100 cm^{-1}, which were completely absent in the C-HyG spectrum, indicating the formation of a hydrazone bond.

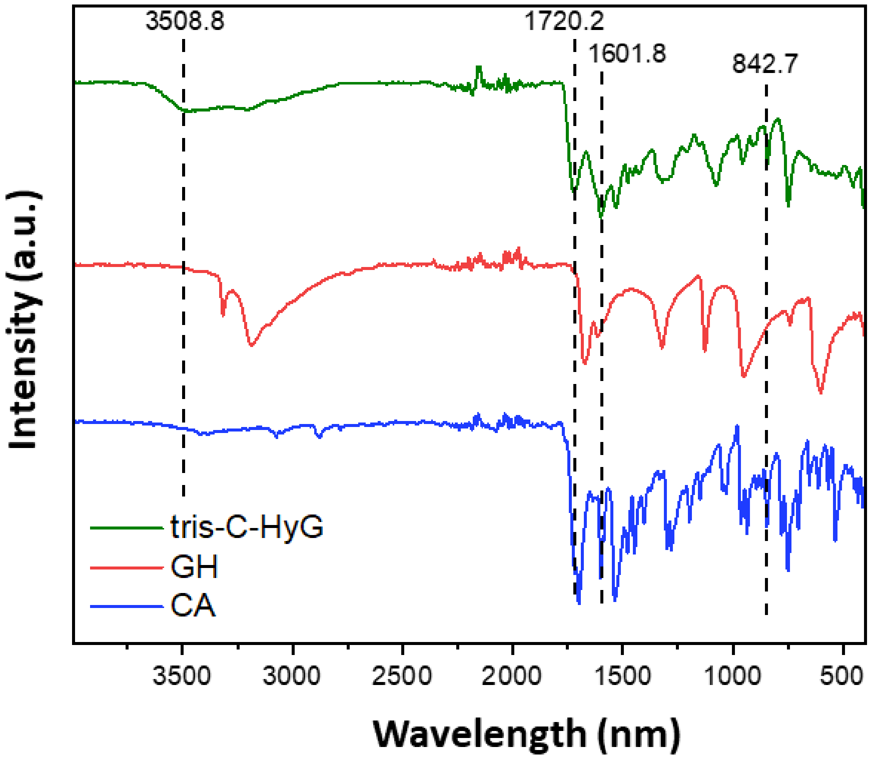

Figure S3: *Stacked FTIR spectra of the precursors* ***CA, GH*** *and hydrogelator* ***C-HyG***.

- **Gelation properties**
- **Effect of co-solvent**

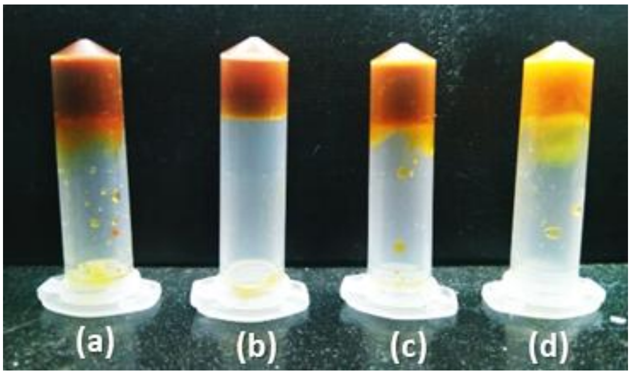

Figure S4*: in-situ hydrogelation of* ***C-HyG*** *in PBS buffer (at pH 5) with (a) DMF; (b) THF; (c) MeOH and (d) DMSO in 1:1 ratio.*

Table S1: *Gelation time upon mixing GH (30 mM) with CA (90 mM) dissolved in various co-solvents*

Sl No.	Solvent	Time (minutes)
1	DMF	10
2	THF	10
3	DMSO	10
4	MeOH	50

- **Determination of sample composition using LC-MS**

Table S2: *Relative abundance of different products formed from hydrazone reaction between CA and GH obtained by LC-HRMS analysis*

Major product	Relative abundance in MeOH (%)	Relative abundance in DMF (%)	Relative abundance in THF (%)	Relative abundance in DMSO (%)
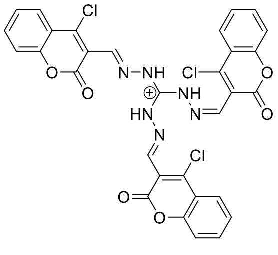 **C-HyG**	63.4 %	87.3	90.03	-
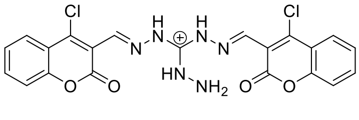 **bis-C-HyG**	29.2 %	11.9	7.8	99.3
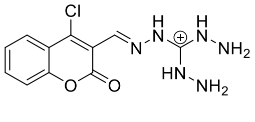 **mono-C-HyG**	7.3 %	0.8	1.2	0.3
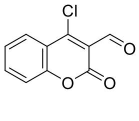 **CA**	-	-	-	0.6

- **Determination of minimum gelation Concentration (MGC)**

Table S3: *MGC in different co-solvents.*

Solvent	DMF	THF	DMSO	MeOH
Concentration of **CA** (in mM)	66	52.5	66	66
Concentration of **GH** (in mM)	22	17.5	22	22

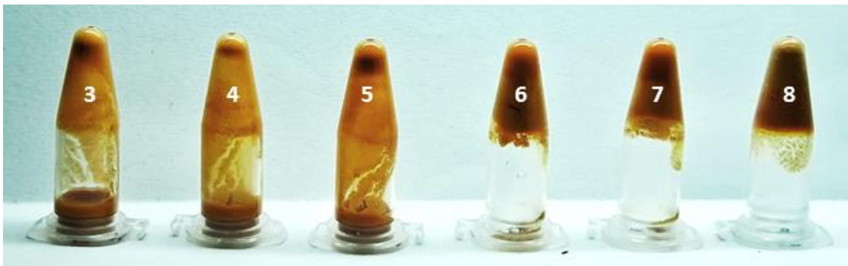

Figure S5*: Determination of MGC via inverted vial test using MeOH as a co-solvent (The number on the vials denotes the serial no. in* ***Table S4****)*

Table S4: *Determination of the MGC at pH = 5 in MeOH as a co-solvent, varying the concentration of the building blocks.*

**Serial No.**	**1**	**2**	**3**	**4**	**5**	**6**	**7**	**8**
Concentration of **CA** (in mM)	30	37.5	45	60	63	66	69	75
Concentration of **GH** (in mM)	10	12.5	15	20	21	22	23	25
Observation	No gel formed	No gel formed	No gel formed	No gel formed	No gel formed	**Gel**	**Gel**	**Gel**

- **Rheological analysis**

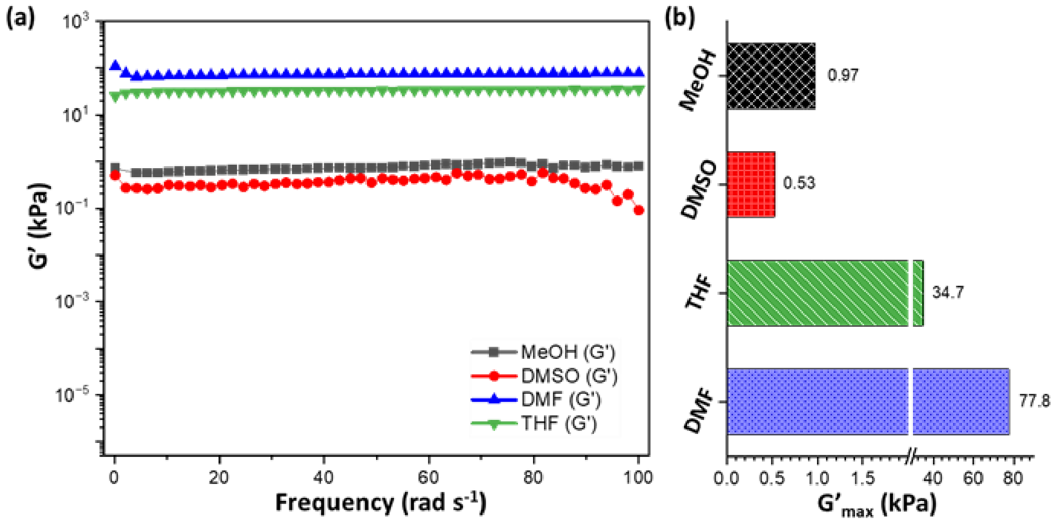

***Figure S6**: (a) Frequency sweep of **C-HyG** hydrogels in different co-solvents at pH 5.0 under isostrain condition; (b) maximum G' observed with different co-solvents.*

- **Kinetic Analysis**

The rate constant for **C-HyG** formation was determined by mixing GH solution (30.0 µM) in PBS buffer (pH 5.0, 6.0 or 7.0) with a methanolic solution of CA (90.0 µM), maintaining a constant 1:1 (v/v) buffer and methanol across all the experiments. The reactive groups (hydrazide from GH and aldehyde from CA) were kept at a 1:1 molar ratio to ensure second-order reaction kinetics. Product formation was monitored by measuring absorbance at 350 nm at regular intervals using a JASCO V-780 UV–Vis spectrophotometer. The second-order rate constant (k) was calculated using a second-order rate equation (Equation 1).

$$\frac{1}{[A_o]-[P]} = kt + \frac{1}{[A]}$$ ....**Equation 1**

Where, $[A_0]$ = Initial concentration of GH, [P] = Concentration of product formed at time t minutes, and k = second order rate constant

On plotting, $\frac{1}{[A_o]-[P]}$ as the dependent variable and time (t) as independent variable, and using the linear equation (y = mx + c) in Origin 2022, the slope obtained denotes the rate constant k (Table S5). The best fits of hydrazone reaction in between CA and GH are given in Figure S7.

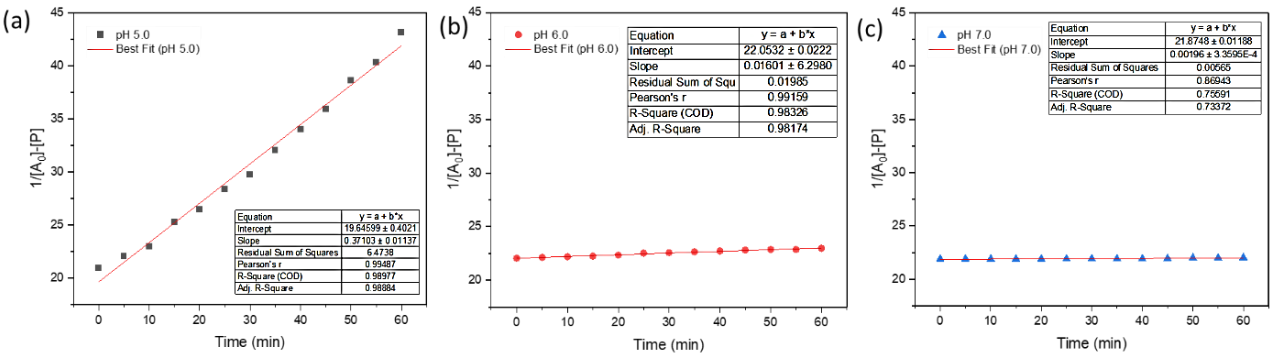

Figure S7: *Best fits for hydrogelation kinetics in PBS buffer at (a) pH = 5.0; (b) pH = 6.0; and (c) pH = 7.0.*

Table S5: *Rate constant for **C-HyG** formation under different pH conditions.*

Sl No.	System	Rate constant, k (L mol^{-1} min^{-1})	k_{rel}	R^2
1	pH 5.0	37.1 x 10^{-5}	185.5	0.99
2	pH 6.0	1.6 x 10^{-5}	8.0	0.98
3	pH 7.0	0.2 x 10^{-5}	1.0	0.76

- **Characterization of hydrogel material via LC-MS**

Table S6: Relative abundances of the products formed from the hydrazone reaction between CA and GH at different pH as determined by LC-MS analysis

Product	Relative abundance in pH 5.0 (%)	Relative abundance in pH 6.0 (%)	Relative abundance in pH 7.0 (%)
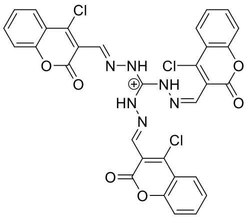 **C-HyG**	63.4 %	28.8	46.0
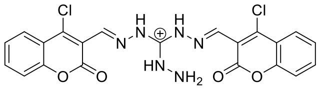 **bis-C-HyG**	29.2 %	36.0	50.4
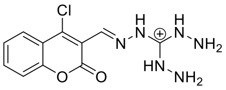 **mono-C-HyG**	7.3 %	35.2	3.6

Table S7: Relative abundances of the products formed by varying concentration of CA and GH at pH = 5.0 as determined by LC-MS analysis

Product	Relative abundance<br>GH= 30 mM<br>CA=90 mM	Relative abundance<br>GH= 30 mM<br>CA=120 mM	Relative abundance<br>GH= 30 mM<br>CA= 150 mM
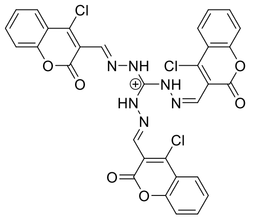<br>**C-HyG**	63.4 %	84.4 %	92.1 %
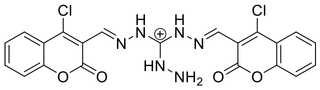<br>**bis-C-HyG**	29.2 %	15.6 %	-
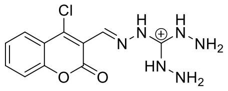<br>**mono-C-HyG**	7.3 %	-	7.9 %

- **Rheology analysis with different precursor composition**

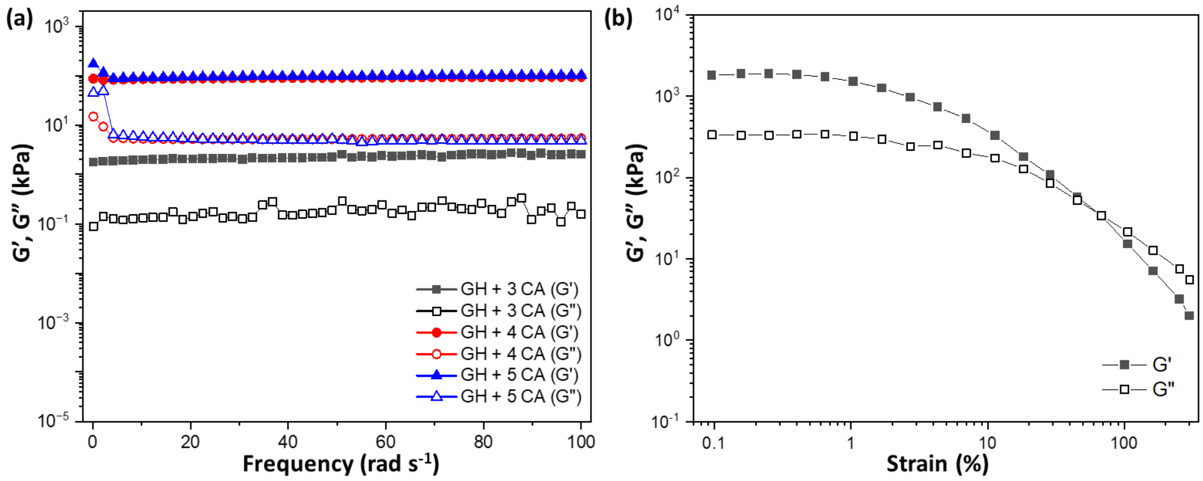

Figure S8: *(a) Frequency sweep of* ***C-HyG*** *hydrogel with different ratio of CA precursor (3-5 eq.) and (b) Strain sweep of* ***C-HyG*** *hydrogel when CA = 3 eq. of GH prepared in methanol and PBS buffer at pH 5.0.*

- **Scanning Electron Microscopy (SEM) Images**

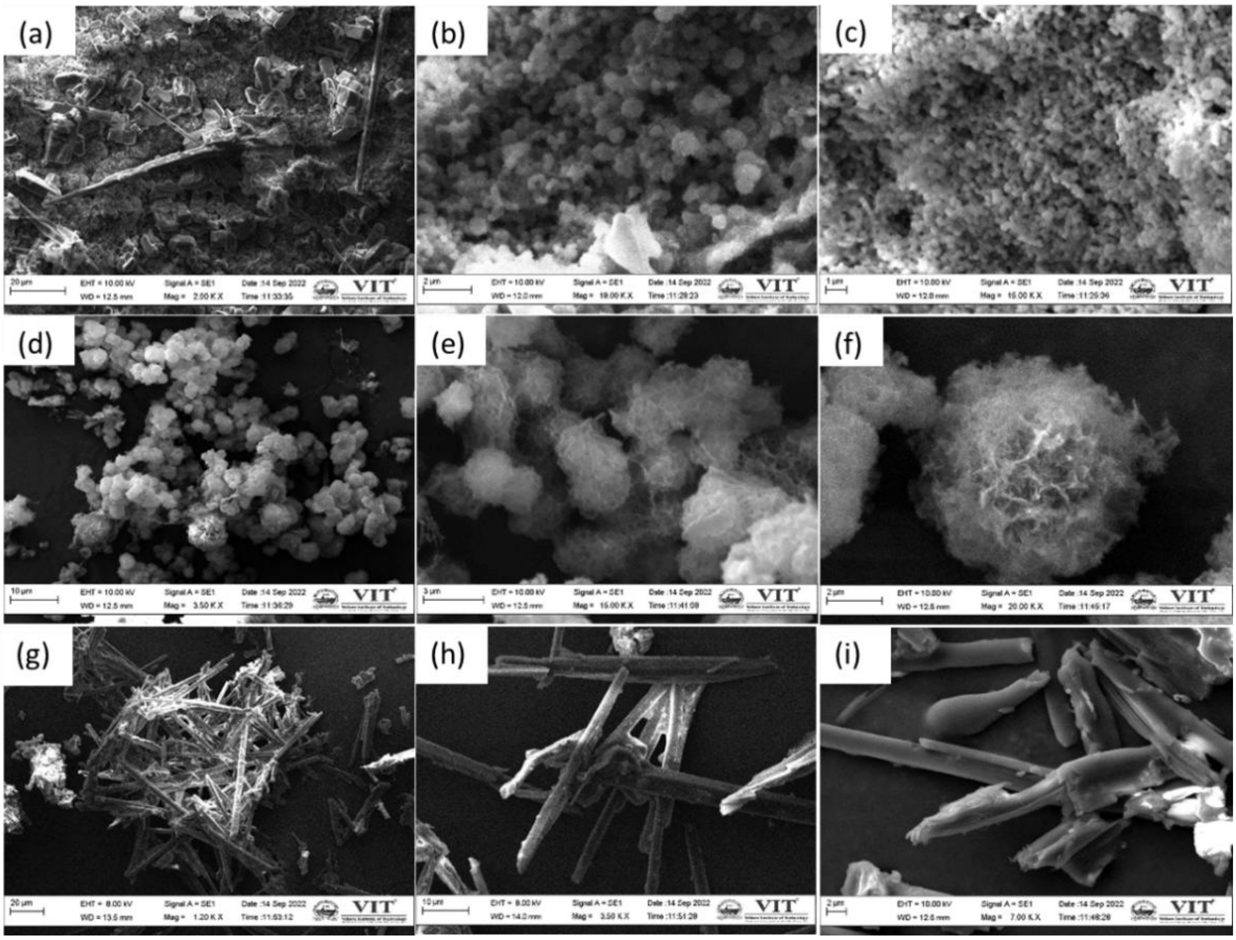

Figure S9*: SEM images of hydrogel material obtained at (a-c) pH = 5, (d-f) pH = 6, and (g-i) pH = 7.*

- ***Field Emission Scanning Electron Microscopy (FESEM) Images***

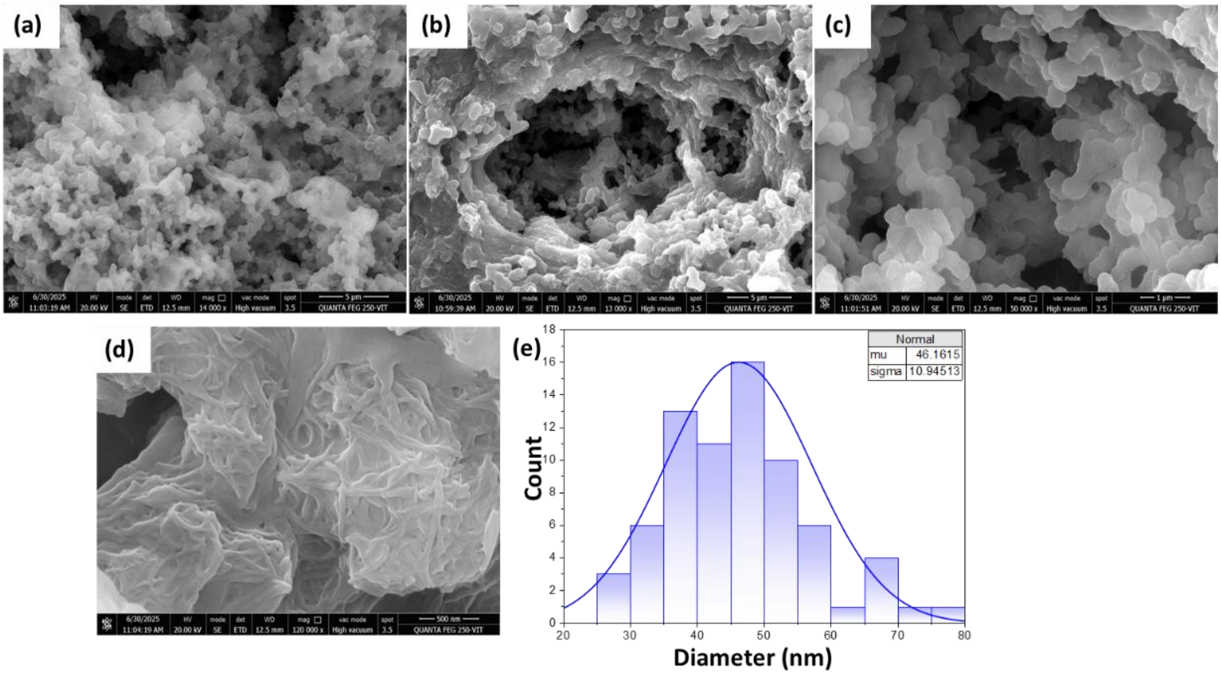

***Figure S10**: (a-d) FESEM images of **C-HyG** hydrogel obtained at pH 5.0, (e) fibre width distribution curve obtained from Figure S10d using ImageJ analysis.*

- **Fluorescence microscopy imaging**

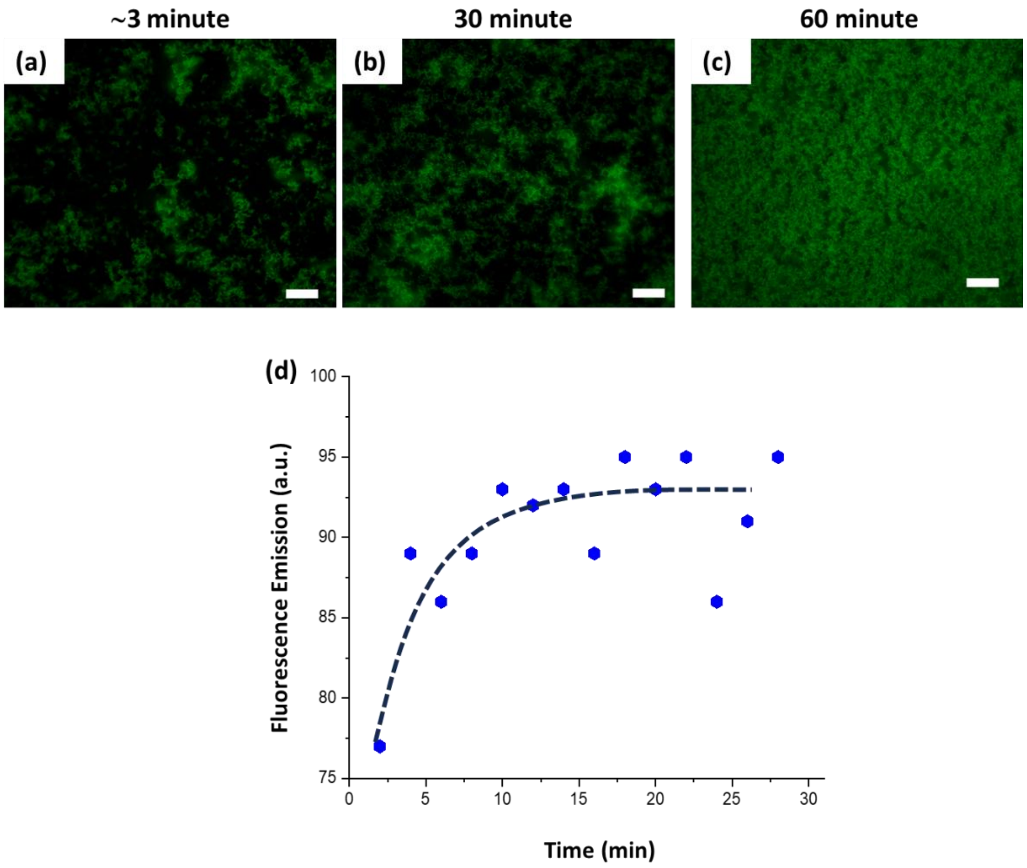

Figure S11: *Fluorescence micrographs of the hydrogel (at pH = 5) captured after mixing CA and GH in stoichiometric ratio at (a) ~3 minute, (b) 30 minutes, and (c) 60 minutes (scale bar: 10 µm); and (d) change in fluorescence intensity as a function of time obtained using ImageJ.*

- **Confocal laser scanning microscopic (CLSM) imaging**

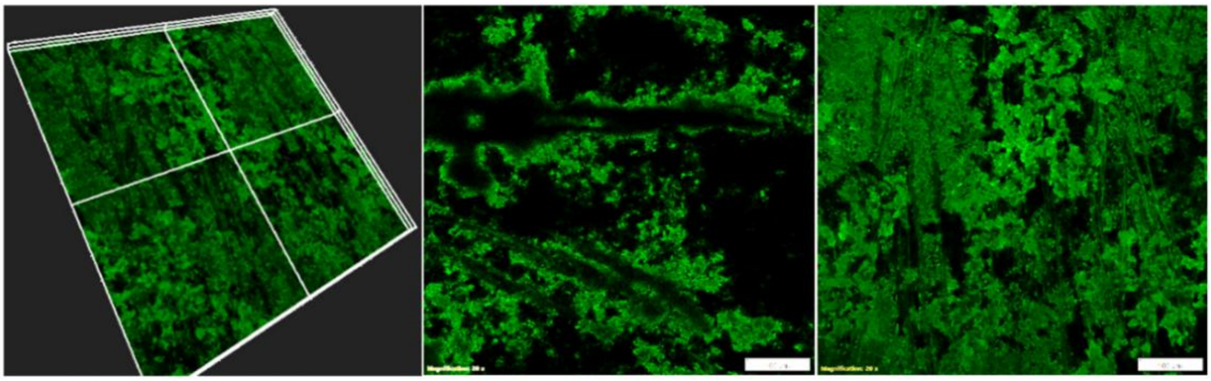

Figure S12: *Hydrogel (at pH = 5) images viewed in CLSM (λ_{ex} = 405nm). Scale bar = 100 µm.*

- **Characterization of C-HyG + Fe(II) complex**
- **FTIR spectra**

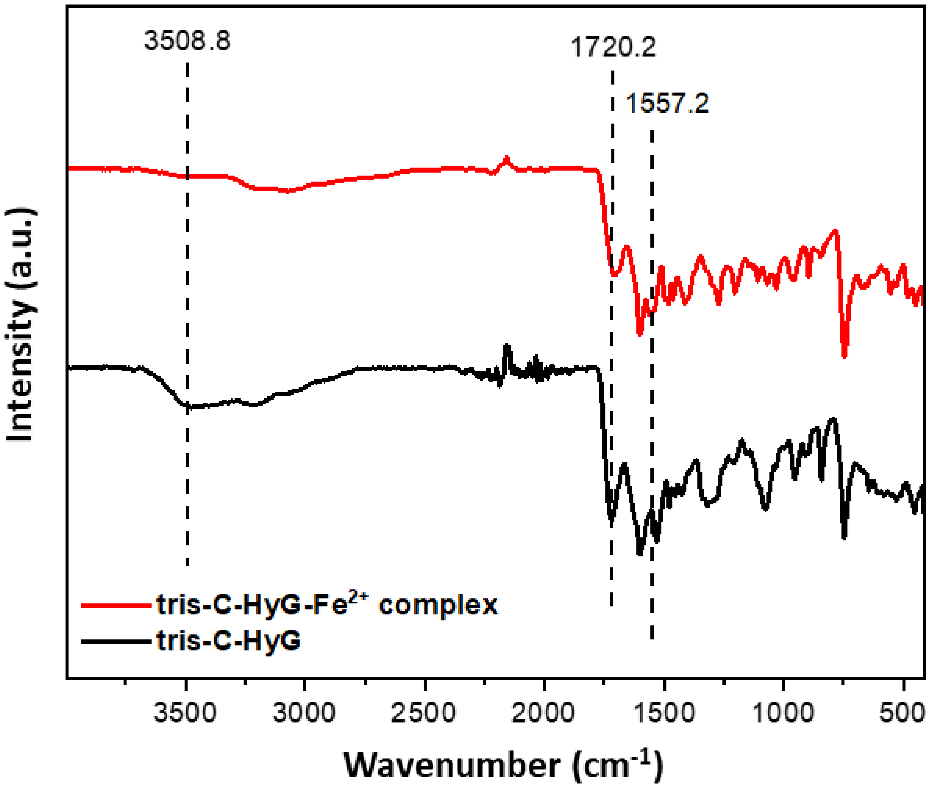

Figure S13: *FTIR spectra comparing the molecular interactions between* ***C-HyG*** *and* ***Fe(II)*** *ions.*

- **X-ray Photoelectron Spectroscopy (XPS)**

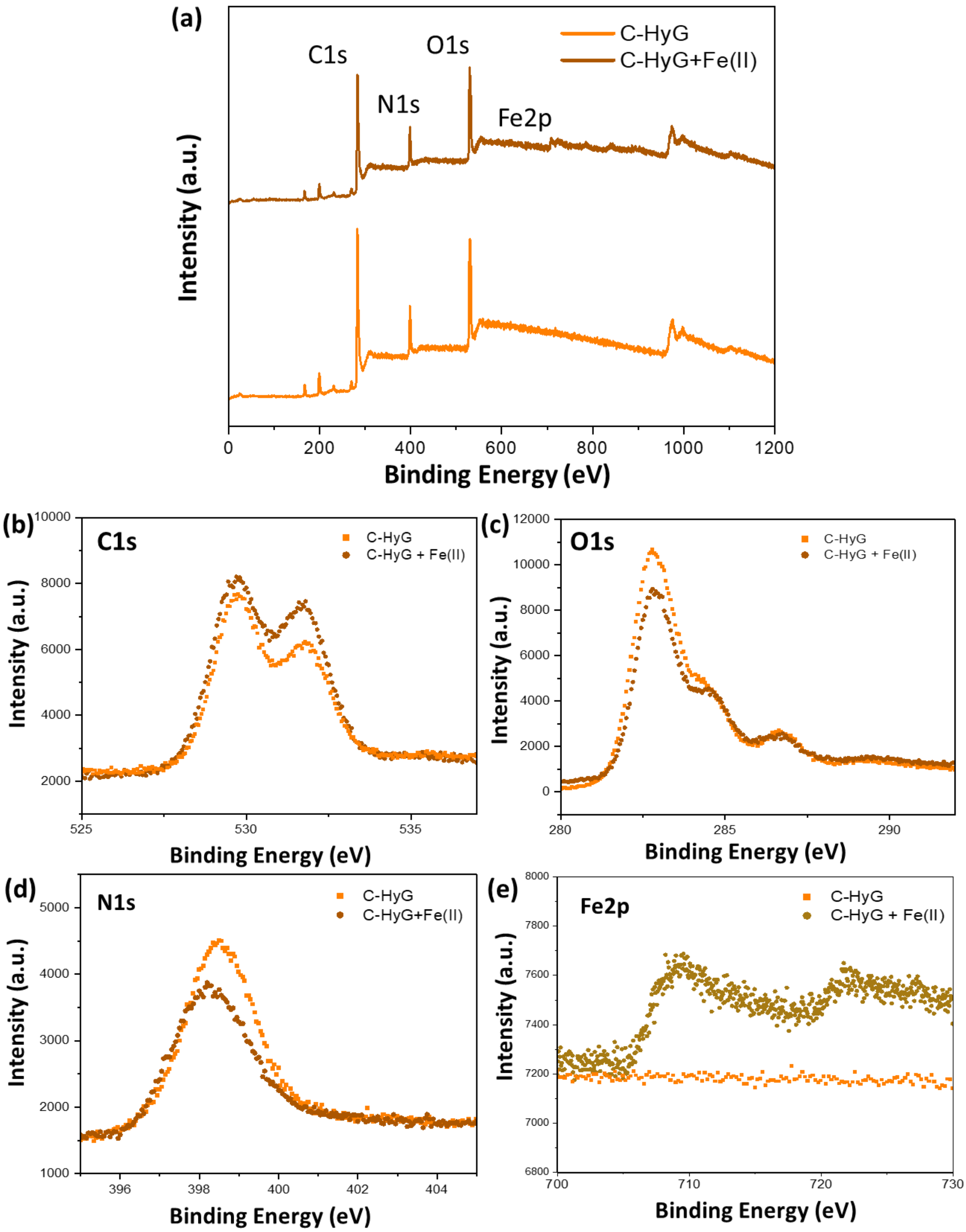

Figure S14: *(a) Comparative XPS spectra of **C-HyG** (orange line) and **C-HyG** with Fe(II) (brown line); (b) shift in intensity of the C 1s energy state; (c) shift in binding energy of the O 1s energy state; (d) shift in binding energy of the N 1s energy state; and (e) Fe 2p energy spectrum in the **C-HyG + Fe(II)** complex, showing the **Fe(II)** binding energy at 209.15 eV.*

- **¹H NMR**

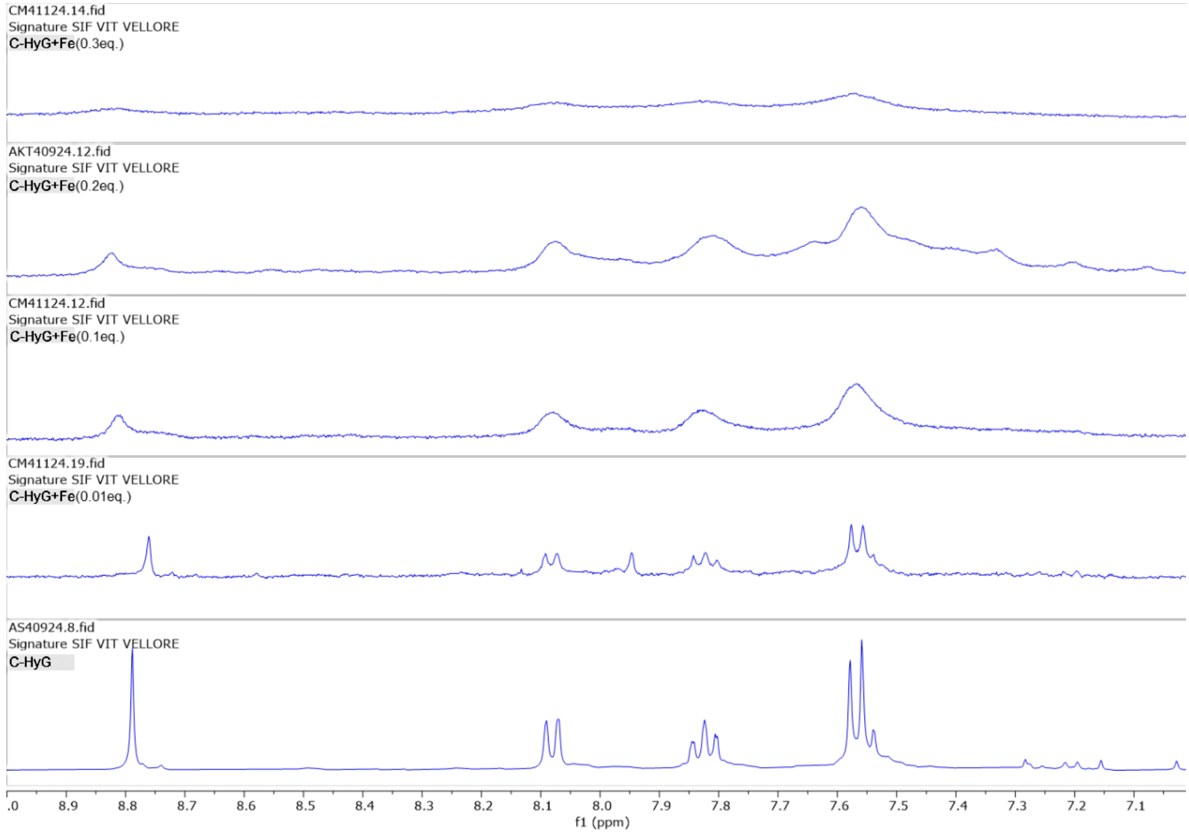

***Figure S15:** Stacked ^{1}H NMR spectrum for comparison of molecular interactions of gelator **C-HyG** with **Fe(II)** conc. ranging from 0 to 0.3 eq.*

- ***Job's plot***

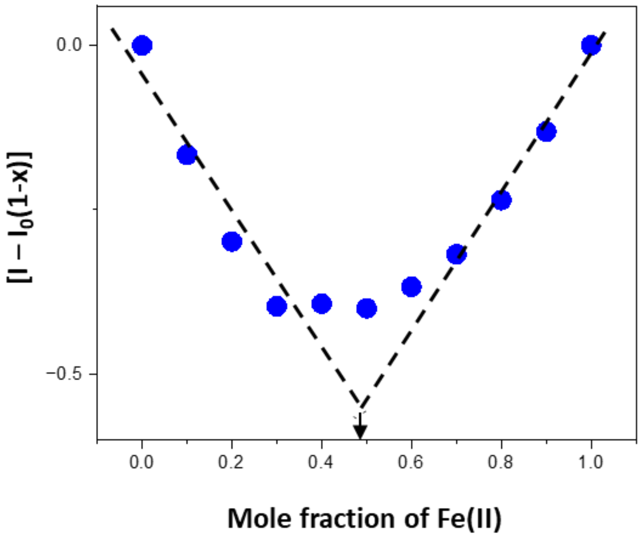

***Figure S16**: Job's plot depicting the stoichiometric coordinating HyG/cation ratio, where intensity refers to $[I-I_0(1-x)]$, I_0 = absorbance of **C-HyG** at 375 nm, I = absorbance at 375 nm after complexation, and x = mole fraction of **Fe(II)**.*

- ***Plausible structure of the complex***

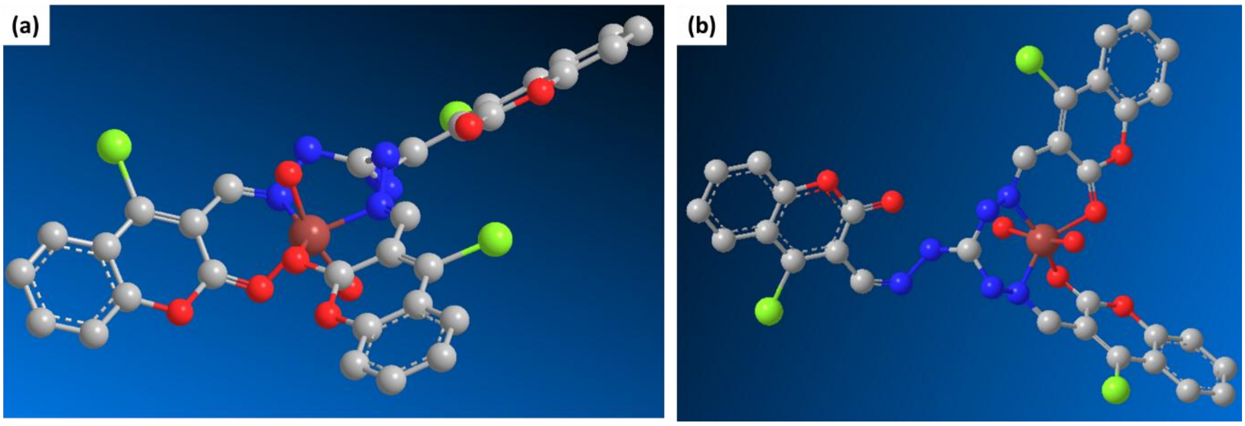

Figure S17: *(a-b) plausible 3D structures of the optimized* ***C-HyG-Fe(II)*** *structure by using Chem 3D software (via MM2 energy optimization).*

- ***HRMS analysis of the complex***

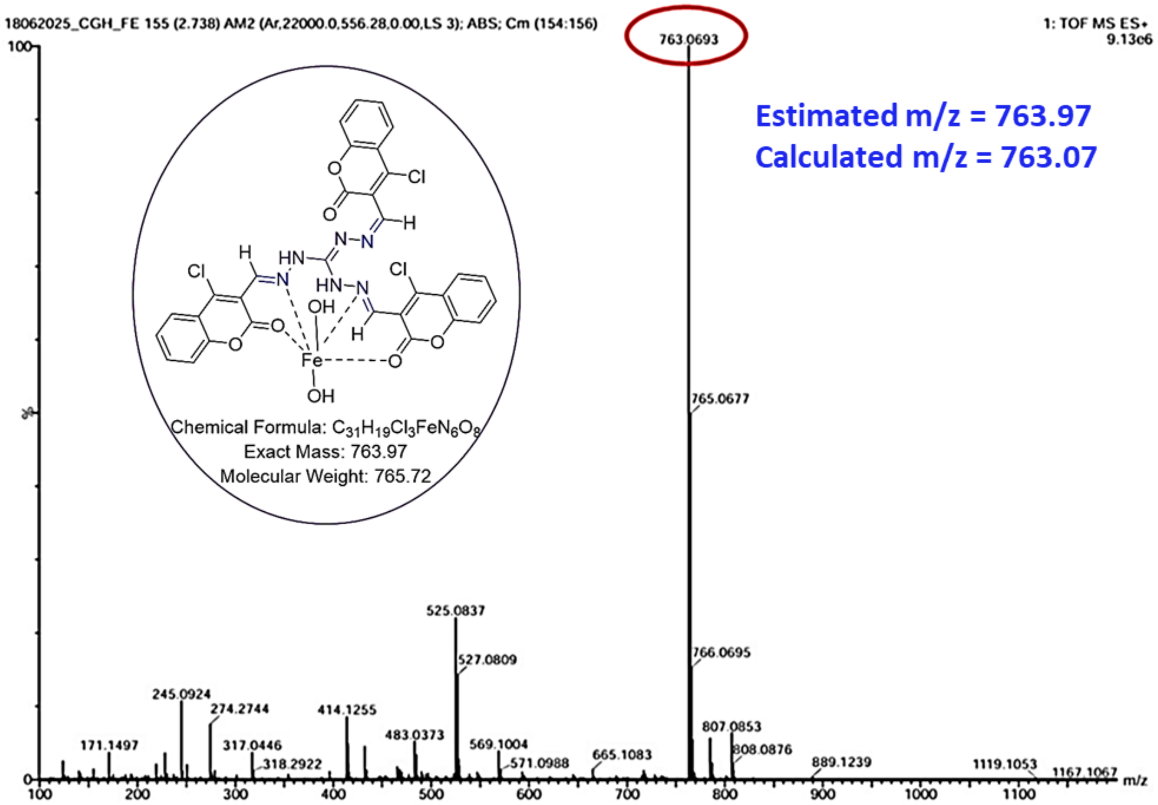

Figure S18: *HRMS spectrum of the* ***C-HyG + Fe(II)***.

- **Limit of detection (LoD)**

The limit of detection was calculated using the standard equation:

$$\boldsymbol{LoD} = \mathbf{3.3} \times \frac{\boldsymbol{Standard\ deviation\ of\ C\text{-}HyG\ in\ sol\ state\ (\alpha)}}{\boldsymbol{Slope\ of\ standard\ curve\ (K)}}$$

Where, α=0.101, and K=0.01037

$\boldsymbol{LoD}$ = 32.14 µM

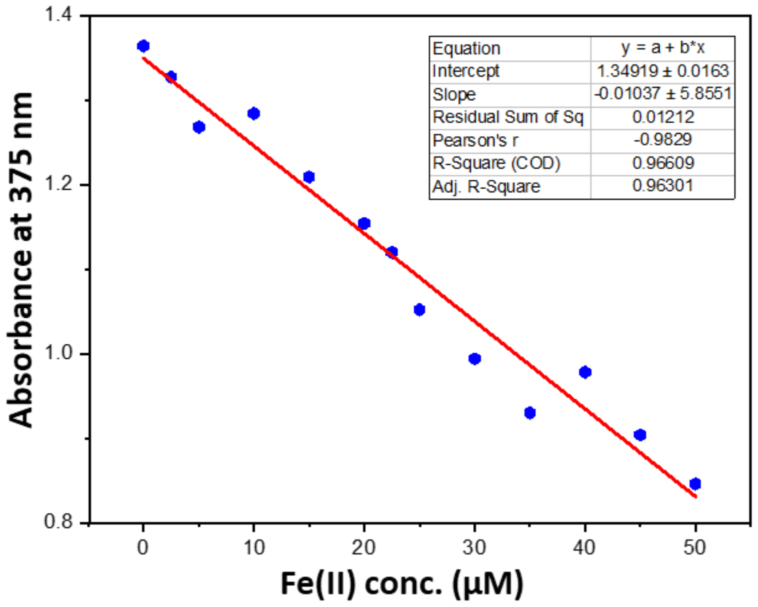

Figure S19: *Plot of absorbance maxima (λ_{max}) with respect to analyte concentration, for determining the LoD using above formula.*

- **pH stability of C-HyG in sol state**

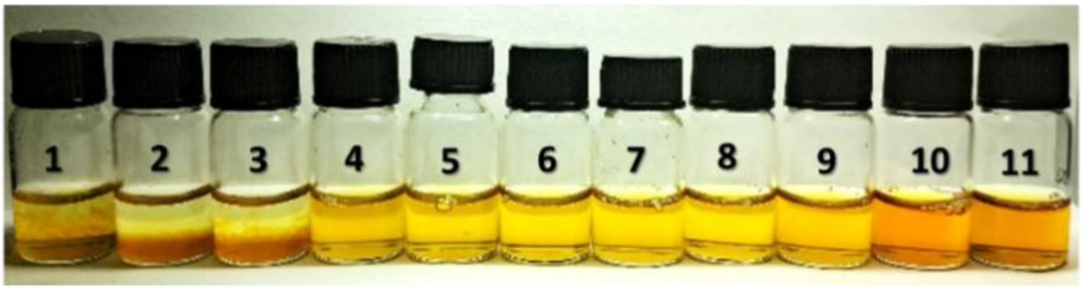

Figure S20: *Effect of pH changes (1-11) of **C-HyG** in sol state.*

- **Reflectance of C-HyG coated paper strips**

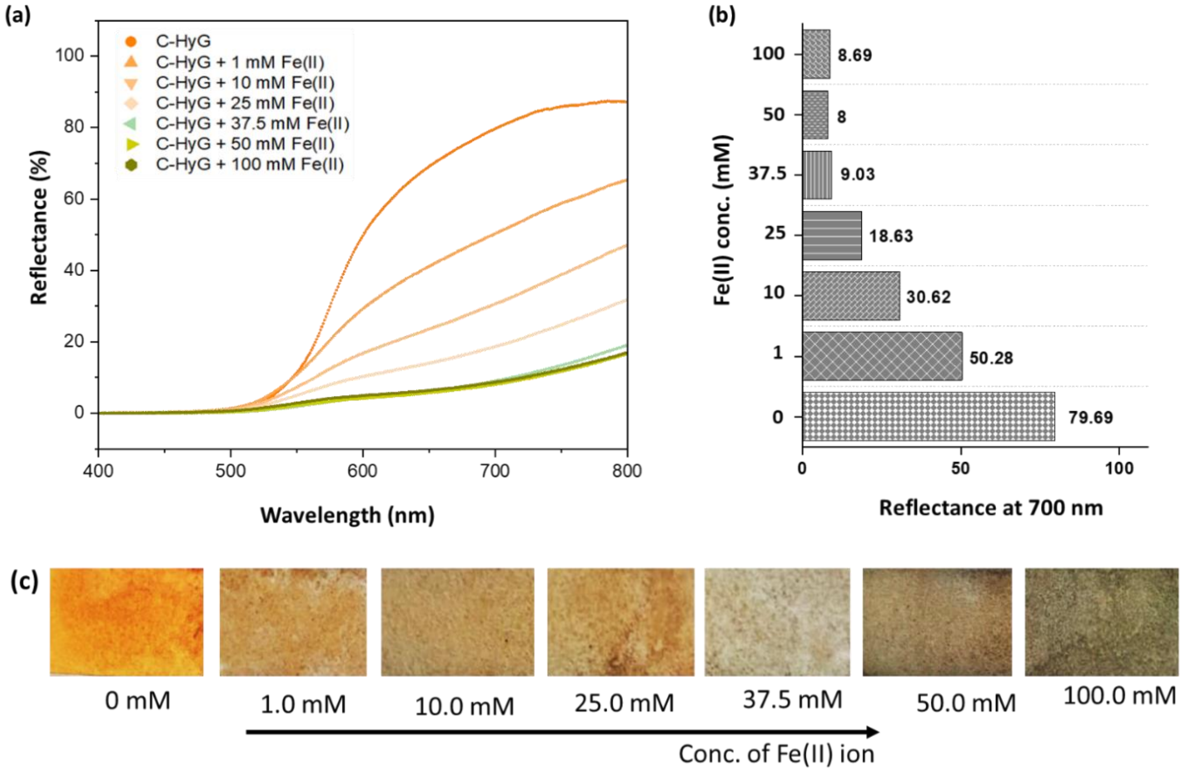

Figure S21: *(a) Reflectance spectrum of* ***C-HyG*** *coated paper strips with different concentrations of Fe(II) ions, (b) reflectance at 700 nm for concentration variance of Fe(II), (c) photographs of paper strips with different Fe(II) concentrations.*

References:

S1. Z. Zhang, X. Dong, J. Yin, Z. -G. Li, X. Li, D. Zhang, T. Pan, Q. Lei, X. Liu, Y. Xie, F. Shui, J. Li, M. Yi, J. Yuan, Z. You, L. Zhang, J. Chang, H. Zhang, W. Li, Q. Fang, B. Li, X. -H. Bu, Y. Han. Chemically stable guanidinium covalent organic framework for the efficient capture of low-concentration iodine at high temperatures. *J. Am. Chem. Soc.* **2022**, *144*, 6821–6829. (https://doi.org/10.1021/jacs.2c00563)
