# Supplementary material for: Catalytically controlled formation of coumarin-based hydrogelator enables colorimetric ferrous ion detection in sol and hydrogel
Source: Commun Chem. 2025 Nov 26;8:372. doi: 10.1038/s42004-025-01760-3 (PMC12658006; doi:10.1038/s42004-025-01760-3)
Supplement: Supplementary file 3 — Description of Additional Supplementary Files [file 42004_2025_1760_MOESM3_ESM.docx]

**Description of Additional Supplementary Files**

File name: Supplementary Video V1

Description: Preparation of coumarin-based hydrogel at room temperature via mixing approach

File name: Supplementary Video V2

Description: Formation of hydrogel network monitored by fluorescence microscope

File name: Supplementary Video V3

Description: A complexation model for Fe(II)-complex.

File name: Supplementary Data 1

Description: NMR spectra of the hydrogelator and for NMR titration with Fe(II)
