# Supplementary material for: Catalytically controlled formation of coumarin-based hydrogelator enables colorimetric ferrous ion detection in sol and hydrogel
Source: Commun Chem. 2025 Nov 26;8:372. doi: 10.1038/s42004-025-01760-3 (PMC12658006; doi:10.1038/s42004-025-01760-3)
Supplement: Supplementary file 7 — Supplementary Data 1 [file 42004_2025_1760_MOESM7_ESM.docx]

**^1^H NMR** (400 MHz, DMSO) δ = 8.47 (s, 3H, C-H associated in hydrazone bond), 8.05 (d, *J* = 7.8 Hz, 3H), 7.76 (d, *J* = 7.9 Hz, 1H), 7.52 (d, *J* = 6.7 Hz, 6H).

***Figure S1****: ^1^H NMR spectra of gelator* ***C-HyG*** *in DMSO-D_6_.*

**^1^H NMR** (400 MHz, DMSO)


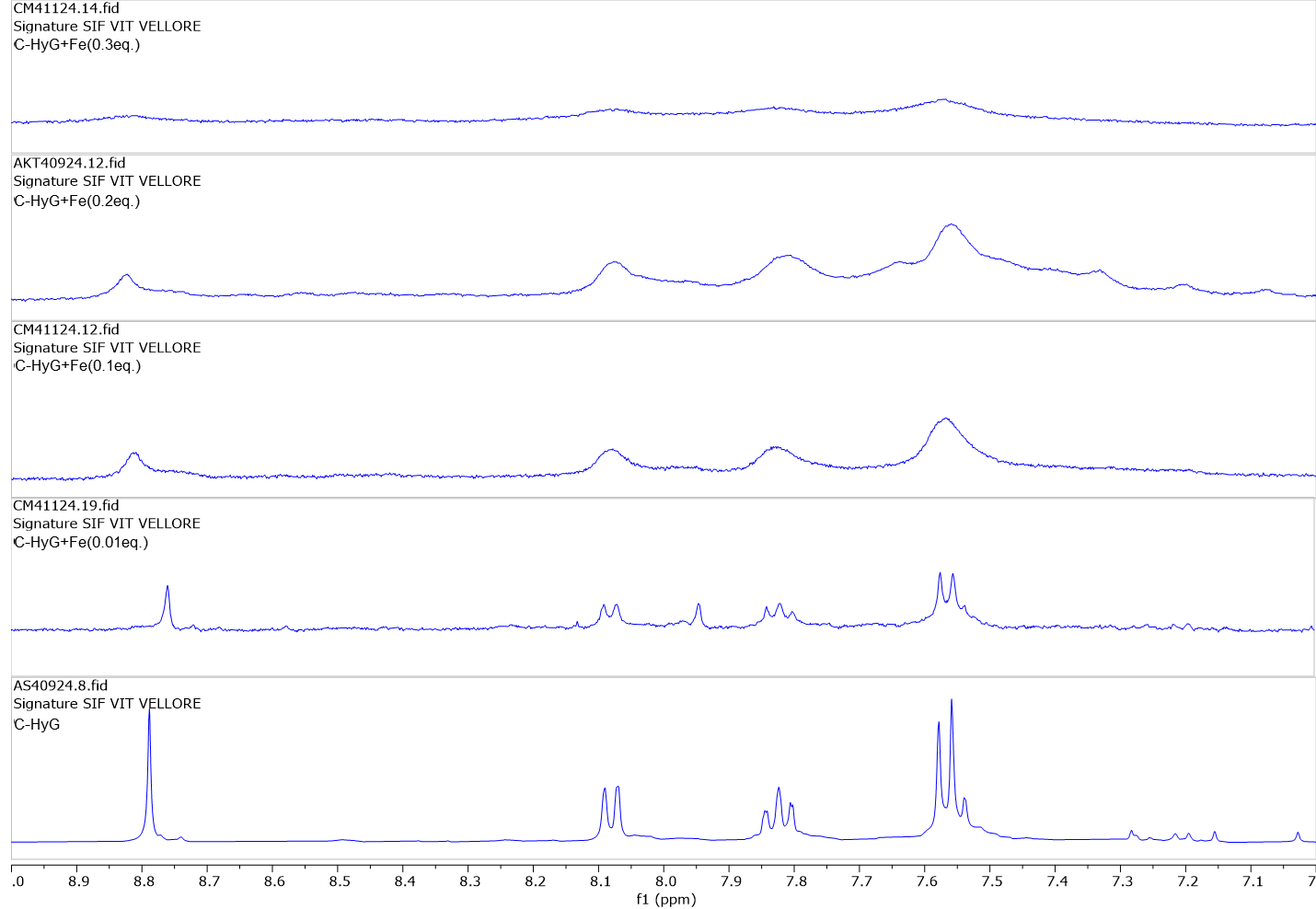


***Figure S2:*** *Stacked* *^1^ H NMR spectrum for comparison of molecular interactions of gelator* ***C-HyG*** *with* ***Fe(II)*** *conc. ranging from 0 to 0.3 eq.*
